# Luteolin Inhibits Behavioral Sensitization by Blocking Methamphetamine-Induced MAPK Pathway Activation in the Caudate Putamen in Mice

**DOI:** 10.1371/journal.pone.0098981

**Published:** 2014-06-05

**Authors:** Tinglin Yan, Lu Li, Baiyu Sun, Fei Liu, Peng Yang, Teng Chen, Tao Li, Xinshe Liu

**Affiliations:** 1 Department of Forensic Medicine, Xi'an Jiaotong University, School of Medicine, Xi'an, Shaanxi, PR China; 2 The Key Laboratory of Health Ministry for Forensic Science, Xi'an Jiaotong University, Shaanxi, PR China; Xi'an Jiaotong University School of Medicine, China

## Abstract

**Goal:**

To investigate the effect of luteolin on methamphetamine (MA)-induced behavioral sensitization and mitogen-activated protein kinase (MAPK) signal transduction pathway activation in mice.

**Methods:**

Mice received a single dose of MA to induce hyperactivity or repeated intermittent intraperitoneal injections of MA to establish an MA-induced behavioral sensitization mouse model. The effect of luteolin on the development and expression of MA-induced hyperactivity and behavioral sensitization was examined. The expression and activity of ΔFosB and the levels of phosphorylated extracellular signal-regulated kinase 1/2 (pERK1/2), phosphorylated c-Jun N-terminal kinase (pJNK), and phosphorylated p38 mitogen-activated protein kinase (pp38) in the caudate putamen (CPu) were measured by western blot.

**Results:**

Luteolin significantly decreased hyperactivity as well as the development and expression of MA-induced behavioral sensitization in mice. ΔFosB, pERK1/2, and pJNK levels in the CPu were higher in MA-treated mice than in control mice, whereas the pp38 level did not change. Injection of luteolin inhibited the MA-induced increase in ΔFosB, pERK1/2, and pJNK levels, but did not affect the pp38 level.

**Conclusions:**

Luteolin inhibits MA-induced hyperactivity and behavioral sensitization in mice through the ERK1/2/ΔFosB pathway. Furthermore, the JNK signaling pathway might be involved in MA-induced neurodegeneration in the CPu, and luteolin inhibits this process.

## Introduction

Repeated, intermittent administration of addictive drugs (e.g., morphine, amphetamine, cocaine, nicotine, and alcohol) can enhance the locomotor response in experimental animals. The enhancement of behavioral response by repeated drug administration is called behavioral sensitization[1]. Recent studies have demonstrated that behavioral sensitization reflects underlying neuroplastic changes that occur as a result of repeated exposure to drugs of abuse[2]–[3]. Behavioral sensitization may be involved in relapse and in compulsive drug-seeking and drug-taking behavior[4]–[6]. Behavioral sensitization represents a robust form of experience-dependent behavioral plasticity and offers a relatively simple model for understanding the neural mechanisms underlying addiction, including relapse[7]–[10].

The major neuroanatomical substrate of behavioral sensitization appears to be the mesolimbic dopamine system, of which the major components are the ventral tegmental area and its projected regions, including the caudate putamen (CPu)[5], [10]. The CPu, which expresses high levels of dopamine receptors (D1R and D2R) and the *N*-methyl-d-aspartate receptor, is a critical site of synaptic plasticity induced by addictive drugs[10]–[16]. Previous studies have demonstrated that modifications in the CPu are involved in movement initiation, learning of motor patterns, drug-related habit learning, and behavioral sensitization[17], [18].

ΔFosB, a truncated product of *fosB*, is a member of the Fos family of transcription factors (others include c-Fos, FosB, Fra1, and Fra2). ΔFosB is induced in the brain's reward regions by chronic exposure to nearly all drugs of abuse[19]. Once induced, the protein persists for long periods because of its unusual stability[19], [20]. The inducible expression of ΔFosB increases locomotor activity, reward responses, and incentive motivational effects, which may lead to a propensity for relapse even after prolonged periods of withdrawal from addictive drugs. This provides direct evidence that the induction of ΔFosB is both necessary and sufficient to produce sensitized behavioral responses to drugs of abuse, which would be expected to make an individual more vulnerable to addiction[20]–[22]. Nestler et al. have shown that the unusual stability of ΔFosB, partly caused by phosphorylation at its N-terminus by casein kinase 2 (CK2), is the basis of its effects on addiction[23].

The mitogen-activated protein kinase (MAPK) pathway is a key signaling pathway involved in the regulation of proliferation, differentiation, and apoptosis in different cells[24]–[26]. Recent studies suggest that it is composed of the extracellular signal-regulated kinase (ERK), c-Jun N-terminal kinase (JNK), and p38 signaling pathways. The ERK signaling pathway is involved in molecular adaptations and long-term behavioral alterations, including conditioned place preference (CPP) and behavioral sensitization, induced by cocaine or psychostimulants[27], [28]. However, the effects of the JNK and p38 signaling pathways on addiction are not yet clear.

In this study, the effects of luteolin, a CK2 inhibitor, on ΔFosB and the MAPK pathway in the CPu were investigated in mice sensitized by methamphetamine (MA). The results suggest that luteolin attenuates the development and expression of MA-induced behavioral sensitization. The results also suggest that the ERK/ΔFosB signaling pathway mediates the beneficial effect of luteolin on behavioral sensitization induced by MA.

## Materials and Methods

All experiments were carried out in strict accordance with the Guidelines on the Care and Use of Laboratory Animals issued by the National Institutes of Health, USA, and were approved by the Institutional Animal Care and Use Committee of Xi'an Jiaotong University.

### Animals

C57BL/6J mice (male, 18–22 g) were purchased from the Experimental Animal Center of Xi'an Jiaotong University (production license number: SCXK (Shaanxi) 2007-001; license number: SYXK (Shaanxi) 2007-003). Mice were randomly divided into 4 mice/cage and housed under a reverse light cycle (lights on from 7:00 P.M. to 7:00 A.M.) in a climate-controlled colony room (room temperature: 21°C±2°C; humidity: 50%±10%). The animals had access to food and water ad libitum. Two days before the experiments, the mice were adapted to the experimental equipment for 2 h/day. Behavioral testing took place between 8:00 A.M. and 5:00 P.M.

### Drugs

Luteolin (Lu) powder (lot number: 62696-5MG; Sigma, USA) was fully dissolved in 100 µl dimethyl sulfoxide and then diluted with saline to the desired concentration. The solution was always freshly prepared before the experiment. Methamphetamine hydrochloride (batch number: 171212200603; China Pharmaceutical and Biological Products, China) was dissolved in saline and was always freshly prepared before the experiment. Animals in the control group were administered vehicle solution (Veh). All drugs were administered via intraperitoneal (i.p.) injection at a dose of 10 ml·kg^−1^.

### Locomotion

All mice were tested in chambers (43 cm×43 cm×43 cm), and their locomotor activities were determined by a SMART Video Tracking System (version 2.5; Panlab Technology for Bioresearch, Spain) after the injections. The “total distance” is the total distance traversed by a mouse as a result of its horizontal locomotor activity during the recording time. This parameter serves as an overall indicator for the increase in locomotor activity induced by the drugs.

**Figure 1:**
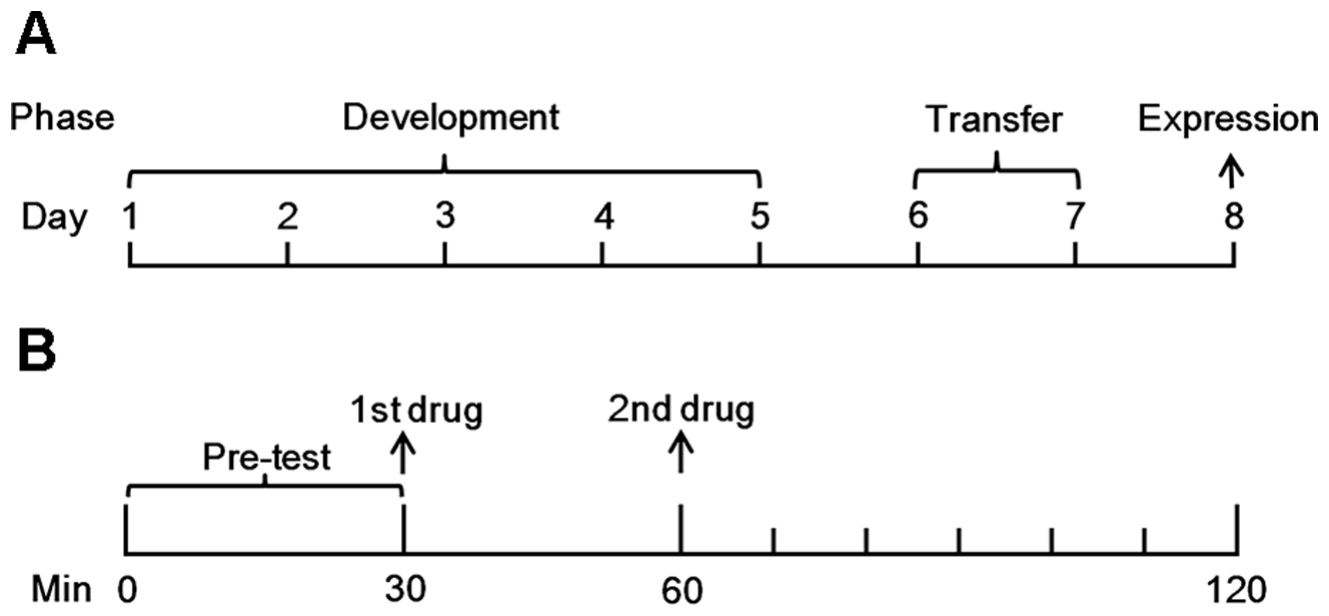
The behavioral sensitization paradigmand dosing schedule. (A) The methamphetamine (MA)induced behavioral sensitization paradigm. On day (D)1-D5,the drugs were injected as schedule. On D6-D7, the drugswere ceased. On D8, the drugs were injected as schedule again. (B)The dosing schedule. Luteolin or vehicle (Veh) wasinjected after pre-testing for 30 min (time point:30 min). MA or Veh was injected after the firstinjections for 30 min (time point: 60 min) andthe test lasts for further 60 min.

The mice (n = 16) were randomly divided into four groups (n = 4/group): control group (Veh+Veh), Lu group (Lu+Veh), Lu and MA combination group (Lu+MA), and MA group (Veh+MA). Lu was administered at 1 mg·kg^−1^, and MA was administered at 2 mg·kg^−1^. After the locomotor activity of the mice in all groups was tested for 30 min, the first drug (Lu or Veh) was injected, and testing continued. After 30 min, the animals were administered the second drug (MA or Veh), and the locomotor activity was monitored for a further 60 min with the total distance recorded every 10 min (Fig. 1B). The same procedures were performed for 5 consecutive days (development phase). Administration ceased on day (D)6–D7 (transfer phase). On D8 (expression phase), all animals were administered the corresponding drugs, and their locomotor activities were measured again (Fig. 1A).

### Western blot analysis

Within 5 min of the completion of the experiment described above, animals were sacrificed, and the CPu was isolated. Brain tissues were instantly frozen and stored at −80°C. A protein extraction kit (Bio-Rad, USA) was used to extract total tissue proteins, and the concentration was measured using the BCA assay. For protein quantification, 50 µg of protein was added to 5× loading buffer, boiled for 5 min, and then subjected to SDS-PAGE. After electrophoresis, the proteins in the gel were transferred to a nitrocellulose membrane, blocked for 1 h with 5% skim milk at room temperature, and incubated in primary antibody at 4°C overnight. We used primary antibodies against ΔFosB (cat number: 2251S; lot number: 2; Cell Signaling, USA), phosphor-ERK (cat number: 4370S; lot number: 7; Cell Signaling, USA), ERK (cat number: 9102S; lot number: 23; Cell Signaling, USA), phosphor-p38(cat number: 4511S; lot number: 10; Cell Signaling, USA), β-actin (cat number: 4970S; lot number: 5; Cell Signaling, USA) at 1:500 dilutions and phosphor-JNK (cat number: 3893-1; lot number: YH122306C; Epitomics, USA), JNK(cat number: 2037-1; lot number: YJ070405CS; Epitomics, USA), p38(cat number: 1544-1; lot number: YE101902C; Epitomics, USA) at 1:1000 dilutions. The next day, the membrane was washed with TBST for 4 times, 10 min each time, and the HRP-labeled secondary antibody (cat number: 31402; lot number: 31460; Pioneer Biology Company, China) at 1:10000 dilution was added for 1 h at 37°C. The membranes were then washed 4 times with TBST, 10 min each time, and developed using the ECL method (Millipore Corporation, USA). A gel image processing system (Bio-Rad, USA) was used to measure the optical density of each band, and the relative expression levels of the proteins of interest were expressed as the AU ratios of pERK1/2/ERK1/2, pJNK/JNK, and pp38/p38.

### Statistical analysis

All data were analyzed using SPSS 17.0 (SPSS, USA). The expression of ΔFosB and the pERK1/2/ERK1/2, pJNK/JNK, and pp38/p38 protein ratios were compared by one-way ANOVA. Locomotor data were analyzed by Student's *t*-test and two-way ANOVA with repeated measures on groups or test sessions. Post hoc multiple comparisons were followed by Student-Newman-Keul tests. “*” and “#” denote P<0.05.

## Results

### The effect of luteolin on MA-induced behavioral sensitization in mice

#### The effect of luteolin on hyperactivity induced by a single dose of MA in mice

**Figure 2:**
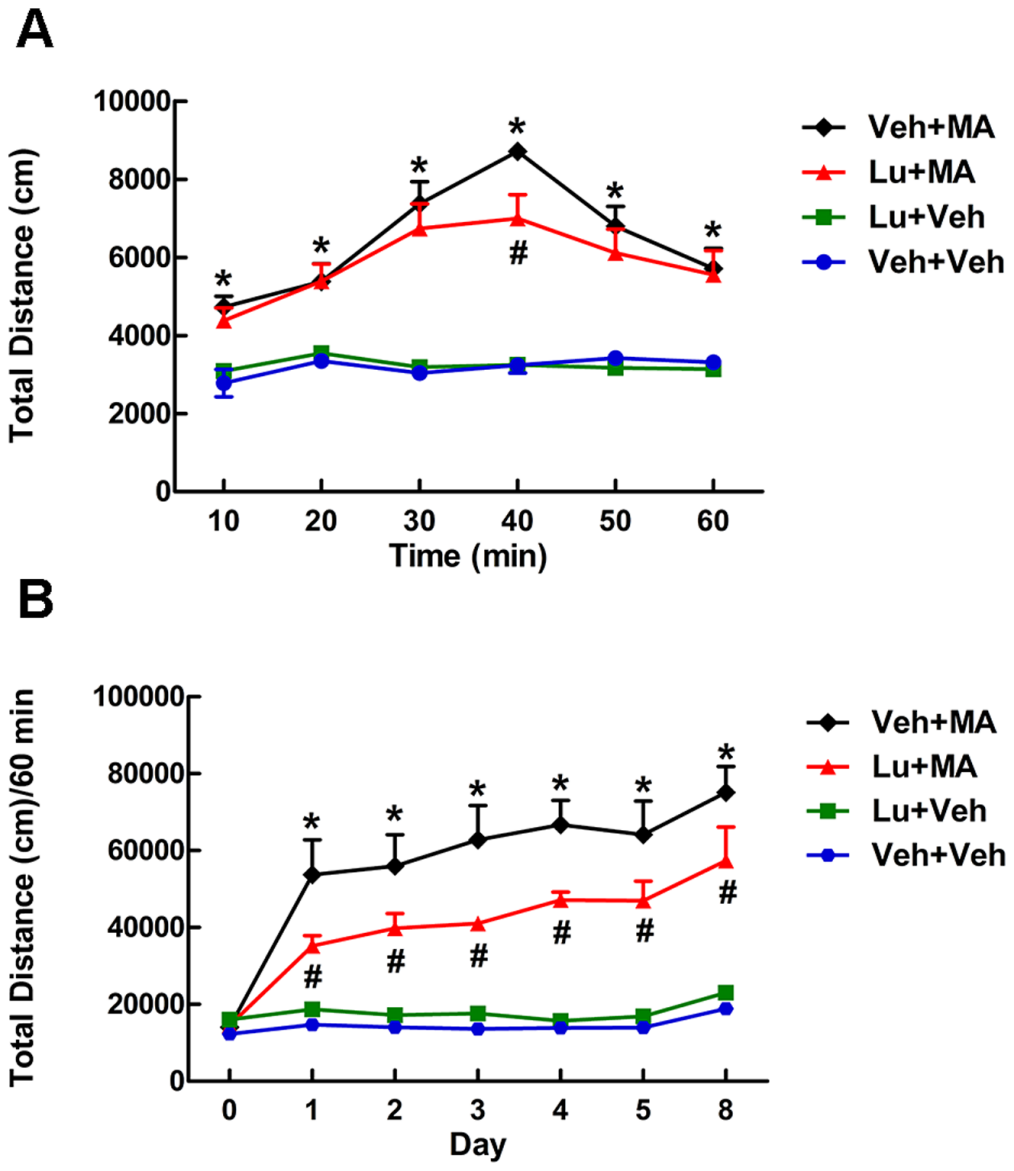
Effect of luteolin on total distance in MA-induced mice. (A) The effect of luteolin (administered 0.5 h before MA injection) on single MA induced mice. (B) The effect of luteolin on the sensitized mice. *P<0.05, compared with the Veh+Veh group. #P<0.05, compared with the Veh+MA group. There was a significant difference between D8 and D1 in the Veh+MA group. Data are presented as the mean ± SEM (n = 4). Data were analyzed using two-way ANOVA and the *t*-test.

Two-way repeated-measures ANOVA revealed a significant main effects of time (F_(5, 60)_ = 22.642, P<0.01), group (F_(3, 12)_ = 37.614, P<0.01), as well as their interaction (F_(15, 60)_ = 7.640, P<0.01) (Fig. 2A). Further multiple comparisons demonstrated, as expected, that a single injection of luteolin (1 mg·kg^−1^) had no significant effect on the locomotor activity of normal mice. As shown in Figure 2A, MA (2 mg·kg^−1^) significantly increased locomotor activity in mice, which peaked at the 30–40 min point (P<0.05 vs. the Veh+Veh control group). A single injection of luteolin (1 mg·kg^−1^) significantly reduced the peak value of locomotor activity acutely induced by MA in mice (P<0.05 at 40 min).

#### The effect of luteolin on the development and expression of MA-induced behavioral sensitization in mice

Two-way repeated-measures ANOVA revealed a significant main effects of time (F_(6, 72)_ = 43.319, P<0.01), group (F_(3, 12)_ = 28.946, P<0.01), as well as their interaction (F_(18, 72)_ = 12.159, P<0.01) (Fig. 2B). Further multiple comparisons found that multiple injections of luteolin (1 mg·kg^−1^) had no statistically significant effect on the locomotor activity of normal mice. On D0, no statistically significant difference in the locomotor activities of the mice in any of the experimental groups was detected. The locomotor activity, as reflected by the total distance, increased from D1 to D5 in both the Lu+MA group and the MA group. However, unlike in the Lu+MA group, in the MA group the total distance on D1 was statistically different from that on D8 (P<0.05). In addition, the total distance in the Lu+MA group was significantly lower than that in MA group from D1 to D8 (P<0.05). This suggests that luteolin (1 mg·kg^−1^) inhibited the development and expression of MA-induced behavioral sensitization in mice.

### The effect of luteolin on MA-induced changes in ΔFosB expression and the MAPK signal transduction pathway in mice

To investigate the mechanisms by which chronic MA administration alters locomotor activity, we examined protein expression in the CPu by western blot.

#### The effect of luteolin on MA-induced changes in the ΔFosB level in mice

**Figure 3:**
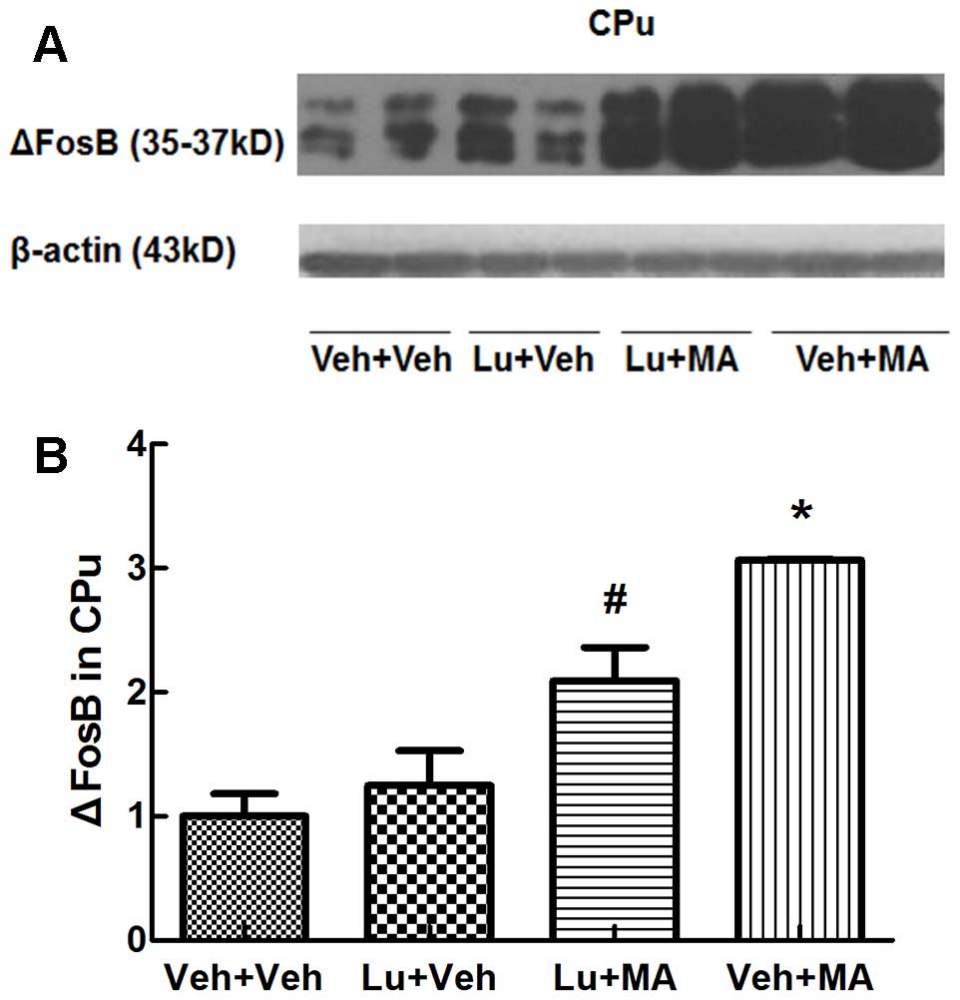
ΔFosB levels in the caudate putamen (CPu) following repeated treatment with methamphetamine. *P<0.05, compared with the Veh+Veh group. #P<0.05, compared with the Veh+MA group. Data are presented as the mean ± SEM (n = 4). Data were analyzed using one-way ANOVA.

Western blot analysis revealed a significant main effect of group (F_(3, 12)_ = 18.832, P<0.01). Chronic administration of MA significantly increased the ΔFosB level in the CPu (P<0.05; Fig. 3). In mice administered Lu+MA, the ΔFosB level was significantly lower than that in mice administered MA alone (P<0.05). Luteolin itself had no statistically significant effect on the ΔFosB level in the CPu.

#### The effect of luteolin on MA-induced changes in the MAPK signal transduction pathway in mice

**Figure 4:**
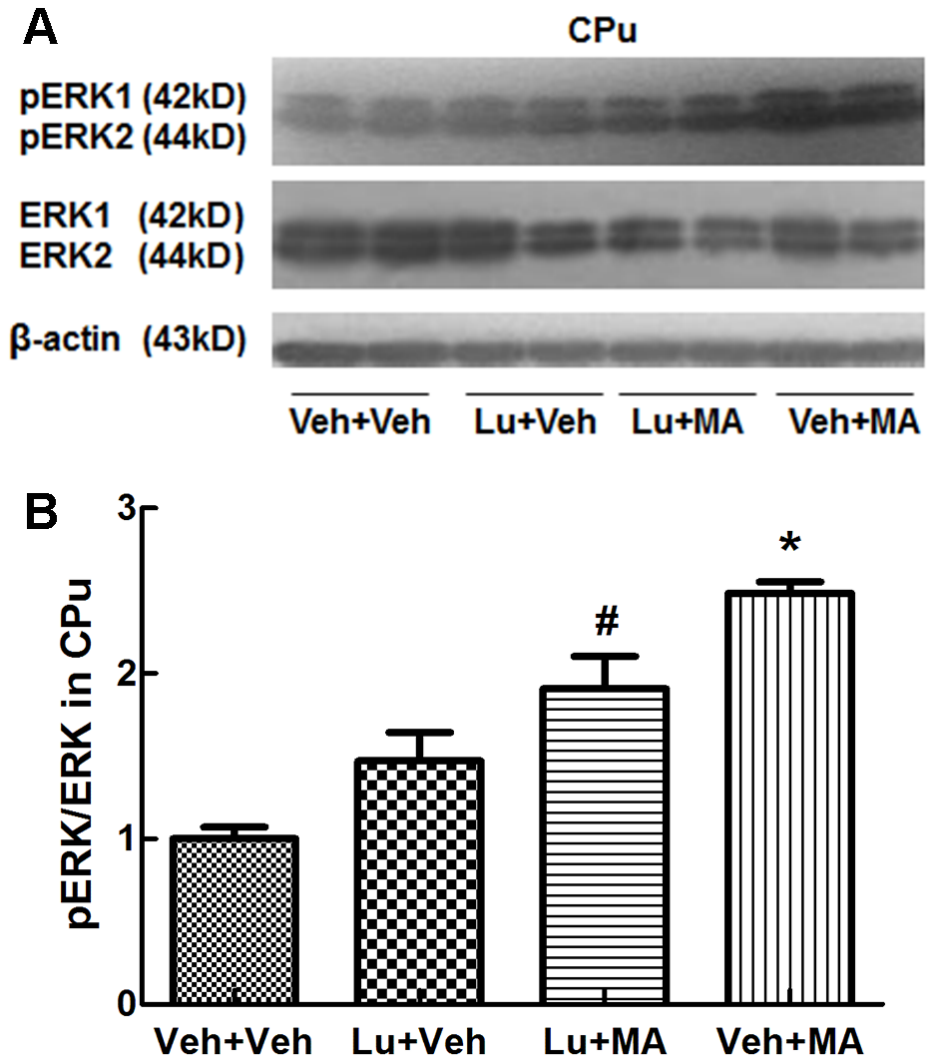
pERK1/2 levels in the CPu following repeated treatment with methamphetamine. (A) Expression of ERK1/2 and pERK1/2 proteins. (B) Ratio of pERK1/2/ERK1/2. *P<0.05, compared with the Veh+Veh group. #P<0.05, compared with the Veh+MA group. Data are presented as the mean ± SEM (n = 4). Data were analyzed using one-way ANOVA.

**Figure 5:**
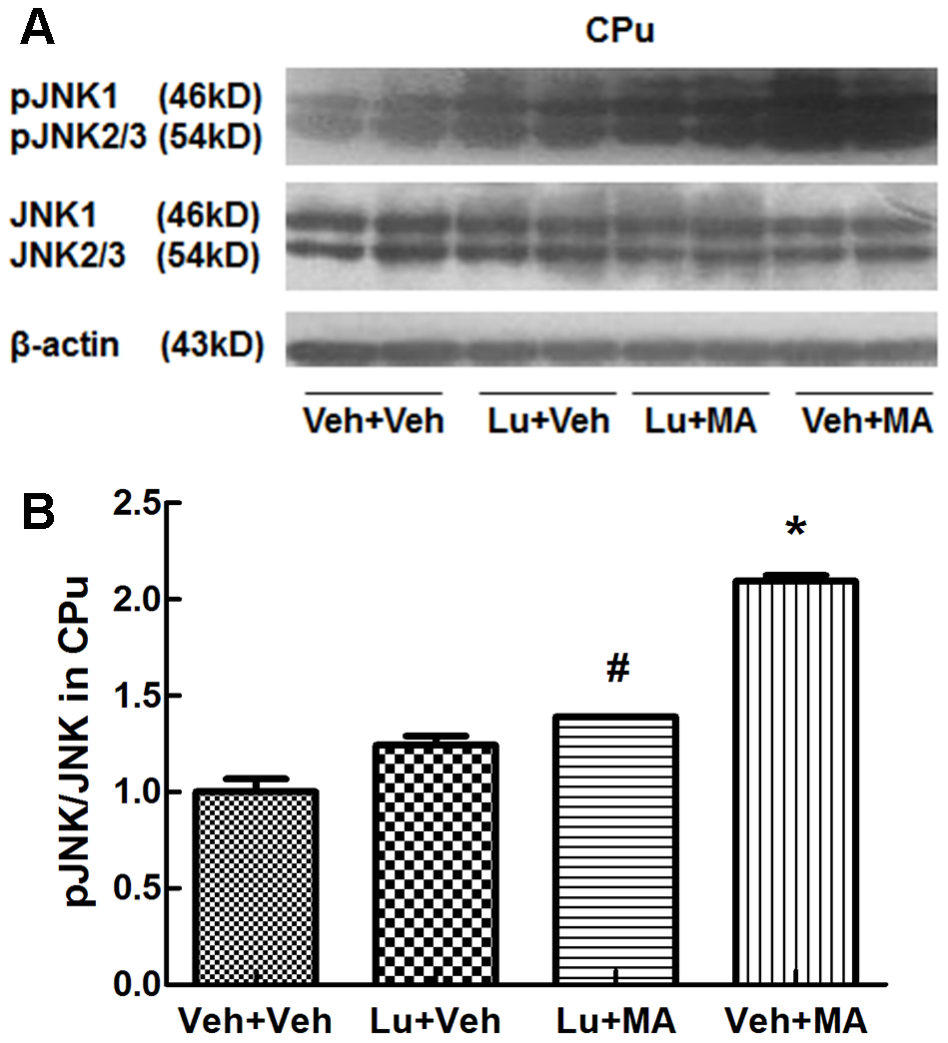
pJNK levels in the CPu following repeated treatment with methamphetamine. (A) Expression of JNK and pJNK proteins. (B) Ratio of pJNK/JNK. *P<0.05, compared with the Veh+Veh group. #P<0.05, compared with the Veh+MA group. Data are presented as the mean ± SEM (n = 4). Data were analyzed using one-way ANOVA.

**Figure 6:**
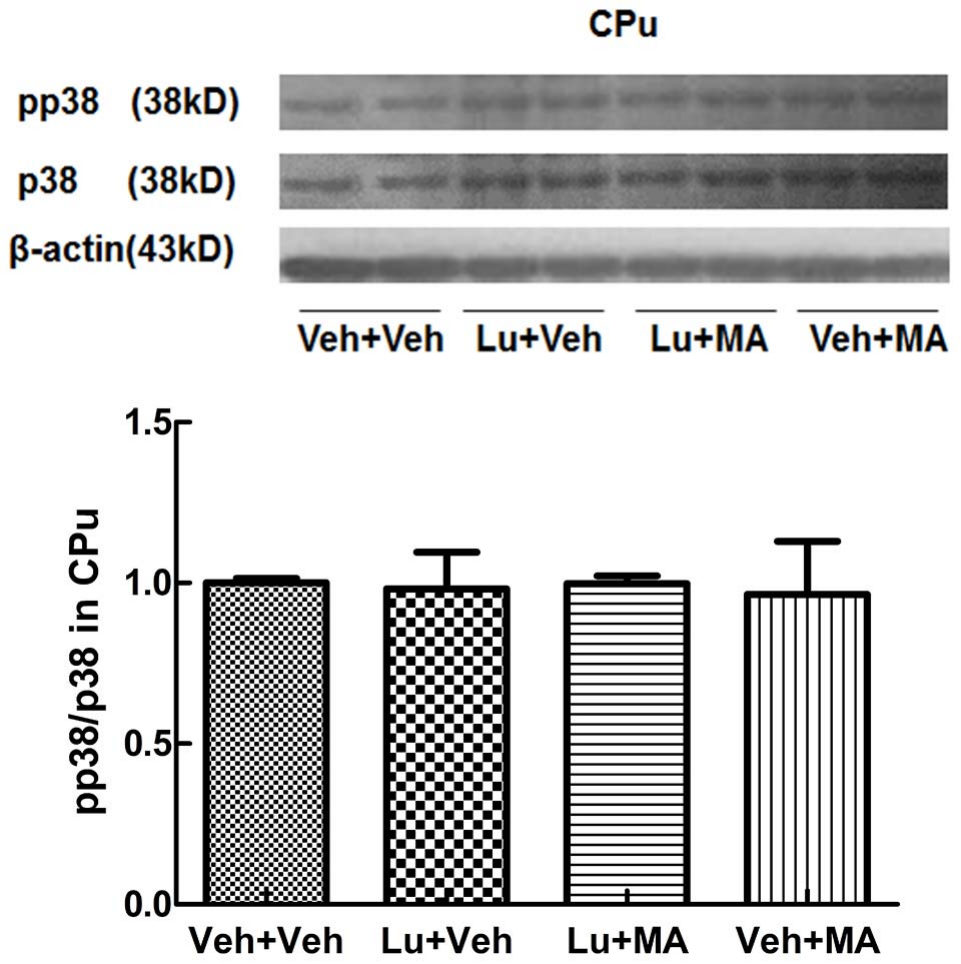
pp38 levels in the CPu following repeated treatment with methamphetamine. (A) Expression of p38 and pp38 proteins. (B) Ratio of pp38/p38. There were no significant differences between the four groups. Data are presented as the mean ± SEM (n = 4). Data were analyzed using one-way ANOVA.

Western blot analysis revealed different main effects of the groups. Specifically, in the CPu, the main effects on pERK1/2/ERK1/2 (F_(3, 12)_ = 20.565, P<0.01) and pJNK/JNK (F_(3, 12)_ = 117.671, P<0.01) were significant, but the main effects on pp38/p38 (F_(3, 12)_ = 0.027, NS) were not remarkable. Further multiple comparisons demonstrated that chronic administration of MA increased pERK1/2 (Fig. 4) and pJNK (Fig. 5) levels in the CPu (P<0.05). The addition of luteolin attenuated the increases in pERK1/2 and pJNK induced by MA (P<0.05). MA did not affect the level of pp38 kinase (Fig. 6). Luteolin itself had no statistically significant effect in any experiment.

## Discussion

Many studies have shown that the repeated, intermittent administration of drugs can produce behavioral sensitization. This is manifested by an increase in locomotor activity, rotational behaviors, and stereotyped behaviors[29]. The results of the present study show that multiple, intermittent i.p. administration of MA (2 mg·kg^−1^) induces notable behavioral sensitization in mice, consistent with previous reports[30]. Behavioral sensitization is thought to be relevant to animal addiction, and it has been used extensively as a promising animal model to evaluate the key features of addiction, including relapse and drug-seeking and drug-taking behaviors[31]. However, the specific mechanisms underlying the regulation of behavioral sensitization are still unclear.

To understand the underlying mechanisms, we measured the expression of ΔFosB protein. The results suggested that MA induced the accumulation of ΔFosB protein. ΔFosB is one of the best-characterized transcription factors known to influence the addiction process. ΔFosB dimerizes with JunD to form activator protein-1 (AP-1) transcription factor complexes. AP-1 complexes then bind to AP-1 sites present in the regulatory regions of many genes, including *Cdk5*, which is responsible for dendritic remodeling[19], [32]–[36]. Consistent with our results, accumulating evidence suggests that ΔFosB increases sensitized behavioral responses, reward responses, and relapse to drugs of abuse[20]–[22]. These key features of addiction are related to the unusual stability of ΔFosB protein. The stability of ΔFosB is due to two factors: (a) the absence of two degron domains present in the C-terminus of full-length FosB which induce the rapid degradation of other Fos family proteins and (b) the phosphorylation of ΔFosB by CK2 at a serine residue (Ser27) in its N-terminus[19], [23].

Because the stability of ΔFosB is regulated by CK2 phosphorylation, we investigated the effect of luteolin, a CK2 inhibitor, on ΔFosB protein expression and locomotor activity. As a CK2 flavonoid inhibitor, luteolin decreases the phosphorylation of serine residues and influences processes such as transcriptional regulation and signal transduction in cells[37], [38]. Consistent with previous studies, we found that pre-treatment with luteolin reduced the ΔFosB protein level in the CPu and the locomotor activity of mice sensitized by MA. These finding suggest that ΔFosB mediates the sensitization induced by MA and that luteolin attenuates the expression of ΔFosB and the formation of sensitization.

Increasing evidence indicates that the MAPK signaling pathway is involved in sensitization and the development of neuroplasticity related to the addictive properties of drugs of abuse[27], [28], [39]–[41]. Therefore, we assessed the MAPK pathway, including the ERK1/2, JNK, and p38 pathways, and the effect of luteolin on mice sensitized by MA.

Previous studies showed that the ERK1/2 signaling pathway mediates cell metabolism and proliferation, the regulation of cell excitability, synaptic plasticity, and drug-seeking and relapse behavior and plays a key role in the formation of craving during withdrawal[42]. Furthermore, several lines of evidence implicate ERK1/2 in the psychostimulant-induced expression of immediate early genes (IEGs) and long-term behavioral alterations, including CPP, psychomotor sensitization, and craving after late withdrawal[43]. Consistent with previous reports, our results indicate that ERK1/2 participates in the behavioral sensitization induced by chronic exposure to MA. Nestler et al. have shown that ΔFosB mediates neural and behavioral plasticity related to addiction[44]. Furthermore, ERK1/2 activation may be involved in the induction of ΔFosB expression[45]. Taken together, the evidence suggests that MA activates ERK1/2, which induces the expression of the *fosB* gene and the accumulation of ΔFosB. We used luteolin to decrease the stability of ΔFosB and found that luteolin suppressed the increase in the pERK1/2 level induced by MA in the CPu. These results indicate that MA induces behavioral sensitization in part through the ERK1/2/ΔFosB signaling pathway and suggest the presence of a feedback mechanism in this pathway. However, further experiments are needed to determine whether feedback to the upstream ERK signaling pathway is mediated directly by ΔFosB or indirectly by its target genes.

Interestingly, similar results were observed in the JNK signaling pathway. The JNK signaling pathway mediates cell differentiation and regulates apoptosis, depending on the cellular context[46]. Studies suggest that single large doses or multiple small doses of MA produce long-term toxic effects[47]–[51]. Several studies suggest that reactive oxygen species (ROS) are important players in MA-induced neurodegeneration in the neurites of dopaminergic neurons[52]–[54]. ROS stimulate the JNK signaling pathway; JNK then phosphorylates c-Jun at Ser63 and Ser73 to activate the transcription of AP-1 target genes[47], [52]. This process, consistent with our results, may eventually induce neurodegeneration. Thus, the JNK signaling pathway may mediate neurodegeneration in brain regions related to MA addiction. We found that luteolin suppressed the increase in the pJNK level induced by MA in the CPu. However, circumstantial evidence indicates that CK2 can phosphorylate JNK on Ser407 and Thr404[55]. Therefore, we do not know whether luteolin, as a CK2 inhibitor, suppresses neurodegeneration by inhibiting the phosphorylation of JNK directly or indirectly through ΔFosB. Taken together, our results show that MA might induce neurodegeneration in the CPu through the JNK signaling pathway and that luteolin suppresses this process. Further research is needed to verify that the JNK signaling pathway is involved in regulating the expression of ΔFosB.

Unlike pERK1/2 and pJNK levels, pp38 levels in the CPu did not change when mice were administered MA or Lu+MA. This suggests that the p38 signaling pathway is not involved in behavioral sensitization or the regulation of ΔFosB protein.

## Conclusion

In conclusion, our present study shows that luteolin can attenuate MA-induced behavioral sensitization through the ERK1/2/ΔFosB pathway. Furthermore, the JNK signaling pathway might be involved in MA-induced neurodegeneration in the CPu, and luteolin inhibits this process.
